# Pediatric ventilation weaning

**DOI:** 10.5935/0103-507X.20200061

**Published:** 2020

**Authors:** Murilo Lopes Lourenção, Werther Brunow de Carvalho

**Affiliations:** 1 Department of Pediatrics, Faculdade de Medicina, Universidade de São Paulo - São Paulo (SP), Brazil.

## IMPORTANCE OF MECHANICAL VENTILATION RELEASE

Mechanical ventilation (MV) is a widely used practice among pediatric intensive care units (PICUs) throughout the world. Data from multicenter studies reveal rates of use ranging from 20% to 64%, and MV typically lasts approximately 5 to 6 days.^(1,2)^ The practice of using artificial methods to provide respiratory care is considered a revolution in the care of critically ill patients, reducing their morbidity and mortality.

On the other hand, it is widely known that these tools can cause a variety of possible complications, such as health care-associated pneumonia, upper and lower airway injuries, and cardiovascular instability.^(3-5)^ Moreover, to use this resource, most of the time, it is necessary to resort to sedative and analgesic drugs.^(1)^ Therefore, it is indispensable to interrupt the MV as soon as possible.

When much time is spent recognizing that MV is no longer essential, the risks and costs are increased (up to $2,000 a day).^(6)^ and good medical practice fails. Currently, the duration of weaning comprises approximately 40% of the total MV time.^(7-9)^

However, defining the optimal timing for extubation is extremely important because early onset may increase morbidity and mortality, the length of stay in the PICU, and the chance of reintubation airway injury. Thus, we should consider the risk factors for extubation failure in each population with similar diseases.^(10)^

## WEANING MANAGEMENT - A MULTIPROFESSIONAL ISSUE

So far, decisions about starting weaning, the best time for spontaneous breathing testing and extubation are primarily doctor-centered.^(11-13)^ However, in very busy PICUs, particularly those in highly complex tertiary centers, medical staff are often focused on other activities. We believe that for the patient who is already able to start the process of withdrawal from MV, the process often ends up being delayed, leading to an increased MV duration and increasing the chances of possible complications that can result.

Thus, studies have been conducted to prove that the involvement of a multidisciplinary team (such as respiratory therapists and nurses) improves ventilatory practices and reduces the duration of MV in PICUs.^(7,14-16)^

In some countries in Europe and North America, there has been a tendency to understand the role of the multidisciplinary team in detecting “weanable” patients and in conducting the protocol.^(17-19)^

A recent Brazilian study aimed to understand the role of professionals in the PICU team during the management of patient weaning. The applied questionnaire obtained interesting data that revealed that 80% of the PICUs surveyed had dedicated respiratory therapists and that in almost 70% of the PICUs, weaning was conducted jointly between the medical team and the team of respiratory therapists. In contrast, in only 12% of the PICUs was weaning conducted by the multidisciplinary team (including nurses).^(20)^

This is no longer the reality found in Australia and New Zealand. Another study showed that in the PICUs analyzed, the nursing team was involved in the weaning process in up to 85% of cases and was responsible for detecting the failure of weaning in up to 94% of patients.^(21)^

However, this is not a new observation. In October 2001, the *Langue Francaise Société de Réanimation*, during its 21st Consensus Conference on Intensive Care and Emergency Medicine: mechanical ventilation weaning, presented a cohort study that demonstrated that a nursing protocol for daily assessment of patients able to start weaning was effective in reducing MV times and the ICU stay.^(22)^ Nevertheless, another Australian study published in 2020 showed no reduction in the MV time or reintubation rates after the implementation of a nursing-driven protocol.^(23)^

Those who advocate against protocols guided by other professionals in Brazil point out some of the structural aspects that hinder such protocols. Primarily, the number of professionals is an issue. The Oceania study pointed out the nurse:bed ratio of 1:1 for patients on MV, and this is not the case for Brazilian PICUs. Brazilian law requires the presence of only one nurse for every eight beds and a nursing technician for every two beds.^(24)^ In addition to the number of professionals per bed, in our opinion, there are other barriers to be overcome to achieve this ideal. We believe that for there to be an adequate multiprofessional weaning protocol, sedoanalgesia protocols must be well established as well as multiprofessional daily rounds. Family members must have a complete understanding of the process so that we can avoid family distress and suffering when progressing through the stages of awakening, the spontaneous breathing test and subsequent extubation. In general, we believe that investment in public health education is necessary to be successful in the application of this type of protocol.

## ESTABLISHMENT OF A WEANING PROTOCOL

In 1996, Ely et al. proved that the simple existence of a protocol of daily evaluation of patients to verify the ability to initiate weaning was able to reduce the time of MV and even the tracheostomy rate;^(25)^ subsequently, countless other authors reached the same conclusion.^(11,26,27)^

Studies have shown that the implementation of a weaning protocol reduces its duration and, consequently, reduces ventilation in children;^(28)^ thus, it is part of the recommendations of the Brazilian Consensus on Mechanical Ventilation.^(29)^

However, there is still not enough scientific evidence to support a better standardized technique for weaning. Even the Paediatric Mechanical Ventilation Consensus Conference (PEMVECC) 2017 was not fully committed to providing guidance regarding the optimal timing of initiation, the approach to weaning and the routine use of any extubation readiness testing.^(30-32)^

There are numerous known weaning techniques, such as the once-daily spontaneous breathing trial (SBT), multiple SBTs, gradual reduction of pressure support, gradual reduction of synchronized intermittent mandatory ventilation (SIMV) and gradual reduction of SIMV and pressure support. In adults, for approximately 20 years, daily SBT has been the most common form of ventilatory weaning,^(8,33,34)^ and even mnemonic strategies have already been created in attempts to implement this routine.^(35)^

There are limited data describing ventilation and weaning practices in PICUs across the world. However, a recent European study showed that daily SBT was also the most commonly used weaning strategy in PICUs^(7)^ However, in our opinion, this is still not a reality in Brazilian PICUs. In our view, the strategy of reducing parameters in SIMV mode is still the strategy most commonly adopted by Brazilian pediatric intensivists.

## EFFORTS TOWARDS THE STANDARDIZATION OF WEANING

In light of the preceding description, it is natural that we are compelled to think of strategies that are effective in reducing weaning time, reducing MV time, and therefore reducing all the consequent burdens. However, there are several factors that directly or indirectly influence this strategy, such as fluid overload, excessive sedation, delirium, malnutrition, positive end expiratory pressure, pulmonary hypertension and diaphragm function, among others.

Therefore, when we think of a ventilation weaning protocol, we must have a macroscopic view and think of the patient as a complex being that is exposed to multiple different practices and with a myriad of possible responses to the proposed therapies.

The same patient may fail during the weaning process due to factors related to the bedside assistant team, such as the frequency and intensity at which parameters are reduced, the timing of fitness for spontaneous breath testing, and even the criteria used for diagnosing weaning failure, which can be more permissive or more rigid.

For Hartmann, a good ventilation weaning protocol must have four strong foundations to be considered robust: predetermined rules for reducing ventilator parameters; aptitude criteria for the spontaneous breathing test (SBT); a well-protocoled SBT; and well-established failure criteria.^(36)^

To the best of our knowledge, it has been well established that, in adults, the use of support pressure is the best way to shorten the duration of MV. Thus, in our practice, we have adopted the use of this strategy for the weaning of ventilated children in our PICUs.

In our opinion, the use of support pressure does not worsen the quality indicators of ventilatory therapy and does not increase failure rates, and in addition, it shortens weaning time. Thus, we emphasize that there is no strong evidence in the literature to recommend a single strategy for pediatric ventilatory weaning. The use of supportive pressure as such a strategy is based on the opinion of these authors, which is based on their extensive clinical practice in using this ventilatory mode.

There have been few studies in this area, and we believe that greater attention should be paid to this issue by pediatric MV researchers.
